# Implications of various phosphoenolpyruvate-carbohydrate phosphotransferase system mutations on glycerol utilization and poly(3-hydroxybutyrate) accumulation in *Ralstonia eutropha *H16

**DOI:** 10.1186/2191-0855-1-16

**Published:** 2011-07-13

**Authors:** Chlud Kaddor, Alexander Steinbüchel

**Affiliations:** 1 Institut für Molekulare Mikrobiologie und Biotechnologie, Westfälische Wilhelms-Universität Münster, Corrensstrasse 3, D-48149 Münster, Germany

**Keywords:** *Ralstonia eutropha*, PHB, carbohydrate phosphotransferase system (PEP-PTS), ABC transporter, glycerol

## Abstract

The enhanced global biodiesel production is also yielding increased quantities of glycerol as main coproduct. An effective application of glycerol, for example, as low-cost substrate for microbial growth in industrial fermentation processes to specific products will reduce the production costs for biodiesel. Our study focuses on the utilization of glycerol as a cheap carbon source during cultivation of the thermoplastic producing bacterium *Ralstonia eutropha *H16, and on the investigation of carbohydrate transport proteins involved herein. Seven open reading frames were identified in the genome of strain H16 to encode for putative proteins of the phosphoenolpyruvate-carbohydrate phosphotransferase system (PEP-PTS). Although the core components of PEP-PTS, enzyme I (*ptsI*) and histidine phosphocarrier protein (*ptsH*), are available in strain H16, a complete PTS-mediated carbohydrate transport is lacking. Growth experiments employing several PEP-PTS mutants indicate that the putative *ptsMHI *operon, comprising *ptsM *(a fructose-specific EIIA component of PTS), *ptsH*, and *ptsI*, is responsible for limited cell growth and reduced PHB accumulation (53%, w/w, less PHB than the wild type) of this strain in media containing glycerol as a sole carbon source. Otherwise, the deletion of gene H16_A0384 (*ptsN*, nitrogen regulatory EIIA component of PTS) seemed to largely compensate the effect of the deleted *ptsMHI *operon (49%, w/w, PHB). The involvement of the PTS homologous proteins on the utilization of the non-PTS sugar alcohol glycerol and its effect on cell growth as well as PHB and carbon metabolism of *R. eutropha *will be discussed.

## Introduction

Biodiesel (fatty acid methyl esters) is currently beside ethanol the major renewable energy source for substitution of petroleum. During production of biodiesel glycerol occurs as a main by-product (about 10%, w/w), thus saturating the glycerol market. Due to the huge surplus of glycerol that lowers its value, it is important to enlarge the field of its application e.g. as substrate for microbial growth and production of biodegradable polymers which in turn reduces the high production costs of polyhydroxyalkanoates (PHA) in industrial fermentation processes.

*Ralstonia eutropha *H16 serves as model organism to study the hydrogen-based chemolithoautotrophic metabolism and has a great potential in industrial applications because of its ability to produce different biodegradable thermoplastics (PHAs). *R. eutropha *is a non-pathogenic, Gram-negative, H_2_-oxidizing β-proteobacterium. The tripartite genome consists of two chromosomes and the megaplasmid pHG1, and its nucleotide sequence was published in 2006 and 2003, respectively (Schwartz et al. 2003; Pohlmann et al. 2006). Autotrophic CO_2 _fixation proceeds via the Calvin-Benson-Bassham (CBB) cycle. Organic carbon and energy sources for heterotrophic growth comprise sugar acids, fatty acids, alcohols, tricarboxylic acid cycle (TCC) intermediates and other compounds. The utilization of sugars is restricted to the amino sugar *N*-acetylglucosamine and to fructose. The latter is taken up by an ATP binding cassette (ABC)-type transporter (*frcACB*) and is then metabolized via the Entner-Doudoroff (ED) pathway (Gottschalk et al. 1964). The uptake of *N*-acetylglucosamine in *R. eutropha *is mediated by a sugar-specific phosphoenolpyruvate:carbohydrate phosphotransferase system (PTS^Nag ^consisting of EI^Nag^-HPr^Nag^-EIIA^Nag ^[*nagF*] and EIIBC^Nag ^[*nagE*]) that functions independently from the two general components of the bacterial PEP-PTS, histidine phosphocarrier protein (HPr, *ptsH*) and enzyme I component (EI, *ptsI*). The PEP-PTS is widespread among bacteria and consists of the above mentioned two cytoplasmic energy-coupling enzymes and a range of carbohydrate-specific Enzymes II, which catalyze the phosphorylation and concomitant translocation cascade (Stülke and Hillen 1998; Barabote and Saier 2005; Deutscher et al. 2006). Except for the Nag-specific EIIABC proteins, no further functional EII-homologous proteins (permease components) exist in *R. eutropha *as usual for β-proteobacteria (Cases et al. 2007). Thus, *R. eutropha *H16 harbours a functional PTS^Nag ^and an incomplete PEP-PTS. Based on the results of previous studies (Krauße et al. 2009; Kaddor and Steinbüchel 2011) the genome of *R. eutropha *was investigated *in silico *for the occurrence of PEP-PTS homologous proteins. Seven gene loci were identified to encode for proteins of the sugar transport system (Table 1). The chromosomal context of each of these genes has already been described, and deletion mutants lacking combinations of genes involved in the PEP-PTS or/and fructose-specific ABC transport were previously generated (Kaddor and Steinbüchel 2011). In addition to the general sugar import, the PEP-PTS exhibits regulatory cellular functions and may serve as a linkage between nitrogen and carbon metabolism (Reizer et al. 1992; Kotrba et al. 2001; Commichau et al. 2006; Velázquez et al. 2007; Pflüger and de Lorenzo 2008; Krauße et al. 2009). Besides the carbohydrate-related PEP-PTS, a paralogous nitrogen-related PTS (PTS^Ntr^) exists in many Gram-negative bacteria whose regulatory functions, components and interactions with the PEP-PTS were extensively reviewed recently (Zimmer et al. 2008; Pflüger-Grau and Görke 2010).

**Table 1:** Overview of detected and investigated genes involved in PEP-PTS and fructose-specific ABC-type transport in *R. eutropha *H16

Gene	CDS	Protein annotation
*ptsM*	H16_A0324*	Fructose-specific EIIA^Man ^component
*ptsH*	H16_A0325*	Histidine phosphocarrier protein HPr
*ptsI*	H16_A0326*	Enzyme I component
H16_A2203	H16_A2203*	HPr-related phosphocarrier protein
H16_A0384 (*ptsN*)	H16_A0384*	Nitrogen regulatory EIIA^Ntr ^component; Mannitol/fructose-specific EIIA^Mtl ^component
*nagF*	H16_A0311*	Phosphocarrier protein, *N*-Acetylglucosamine-specific EI^Nag^-HPr^Nag^-EIIA^Nag ^components
*nagE*	H16_A0312*	*N*-Acetylglucosamine-specific EIIBC^Nag ^components
*nagC*	H16_A0313	*N*-Acetylglucosamine-specific outer membrane protein (porin)
*frcA*	H16_B1498	Fructose-specific ABC-type transporter, ATPase component
*frcB*	H16_B1500	Fructose-specific ABC-type transporter, periplasmic component
*frcC*	H16_B1499	Fructose-specific ABC-type transporter, permease component

Data were obtained using the KEGG (Kyoto encyclopedia of genes and genomes) database at GenomeNet (Kanehisa et al. 2002). The CDS designations specify the locus of each gene on the chromosome (term H16_A and H16_B indicate chromosome 1 and 2, respectively).

* Asterisks point to the seven PEP-PTS homologous genes.

Growth on glycerol is not linked to the PEP-PTS and occurs very slowly in *R. eutropha *H16; it leads to strong expression of hydrogenases and enzymes of the CBB cycle, the key components of lithoautotrophic metabolism (Friedrich et al. 1981). Furthermore, gluconeogenetic enzymes as well as increased oxidative stress proteins (ROS) were identified in 2-D gels during growth of *R. eutropha *on glycerol (Schwartz et al. 2009). The three-carbon non-PTS sugar alcohol glycerol is probably transported across the cytoplasmic membrane through facilitated diffusion mediated by the glycerol uptake facilitator protein GlpF (Sweet et al. 1990; Darbon et al. 1999). Two proteins, a glycerol kinase and a glycerol-3-phosphate dehydrogenase, are involved in the phosphorylation of intracellular glycerol to glycerol 3-phosphate and the subsequent conversion to dihydroxyacetone phosphate (Voegele et al. 1993; Schweizer et al. 1997). The latter is either introduced into gluconeogenesis or catabolized through the ED pathway via pyruvate to acetyl-CoA, the precursor for the TCC and for poly(3-hydroxybutyrate), PHB, biosynthesis. In *R. eutropha *like in most other bacteria, this polyester serves as storage for carbon and energy. It is synthesized in the cytoplasm via acetoacetyl-CoA and 3-hydroxybutyryl-CoA using enzymes encoded by *phaA, phaB *and *phaC *under conditions of carbon overflow and nitrogen limitation (Schlegel et al. 1961a; Schindler 1964; Haywood et al. 1988a, b, 1989). PHB is biodegradable and may replace petroleum-derived polyolefins that are widely used e.g. as packaging materials or in medicine (Solaiman et al. 2006). The cost of carbon substrate in large scale PHA production processes can be as high as 50% of the total operating costs (Lee 2006). Abundant raw glycerol may substitute traditionally used carbohydrates in industrial microbial processes and reduce PHA production costs (Murarka et al. 2008; da Silva et al. 2009). The price for crude glycerol is decreasing continuously and amounts currently to 180-220 € per ton (ICIS pricing 2008). Several laboratories investigated the use of glycerol as fermentation substrate for PHA production in different bacteria, e.g. in *Pseudomonas *as well as *Burkholderia *species (Ashby et al. 2004, 2005; Chee et al. 2010; Zhu et al. 2010). Moreover, an attempt to produce PHB by *R. eutropha *JMP134 and a *R. eutropha *mutant (DSM 545) using commercial and waste glycerol as carbon source was already performed (Mothes et al. 2007; Cavalheiro et al. 2009). However, concerning *R. eutropha *strain H16 the use of glycerol as a low-cost substrate for growth and biosynthesis of PHB in combination with the high biotechnological potential of this strain has largely been ignored. The present study describes an extension of our previous study (Kaddor and Steinbüchel 2011). Since we observed the involvement of homologous PEP-PTS proteins in the utilization of non-PTS substrates, the main focus of this article is on the importance of PTS homologous proteins and other proteins involved in the carbohydrate uptake system of *R. eutropha *H16 on the utilization of the slow-growth substrate glycerol, the conversion to PHB, and its effect on carbon metabolism. Furthermore, the use of glycerol as cheap and abundant carbon source for growth of *R. eutropha *with respect to industrial applications e.g. the production of biodegradable polyesters from renewable resources will be discussed.

## Materials and methods

### Bacterial strains, media and cultivation conditions

**Figure 1:**
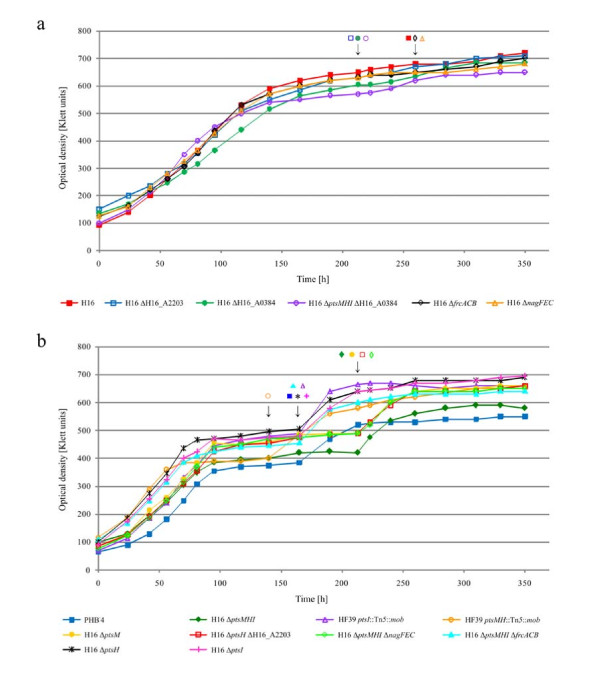
**Growth behavior of *R. eutropha *H16 and various mutants**. Group A comprises the wild type H16, H16 ΔH16_A2203, H16 ΔH16_A0384, H16 Δ*ptsMHI *ΔH16_A0384, H16 Δ*frcACB*, and H16 Δ*nagFEC *(Figure 1a). Group B is represented by mutant strains PHB^-^4, H16 Δ*ptsMHI*, H16 Δ*ptsM*, H16 Δ*ptsH*, H16 Δ*ptsI*, H16 Δ*ptsH *ΔH16_A2203, H16 Δ*ptsMHI *Δ*frcACB*, H16 Δ*ptsMHI *Δ*nagFEC*, HF39 *ptsMH*::Tn*5*::*mob*, and HF39 *ptsI*::Tn*5*::*mob *(Figure 1b). All strains were cultivated under conditions permissive for PHB storage in MSM containing 1.0% (v/v) glycerol as a sole carbon source. Samples were withdrawn in the exponential, early stationary and stationary growth phase to analyze the PHB contents of the cells. After withdrawal of the sample in the early stationary phase, NH_4_Cl was added to the cultures to a final concentration of 0.05% (w/v, arrows). Experiments were done in duplicate.

Bacterial strains used in this study are listed in Table 2. Cells of all strains were cultivated for 20 h in mineral salts medium (MSM) (Schlegel et al. 1961b) containing 1% (w/v) sodium gluconate and 0.1% (w/v) ammonium chloride to promote best growth conditions. After harvesting and washing of the precultures, cells were resuspended in MSM supplemented with 0.05% (w/v) NH_4_Cl and 1% (v/v) of glycerol as a sole carbon source to provide conditions permissive for PHB accumulation and were then incubated for 350 h at 30°C. All liquid cultures were incubated aerobically in baffled Erlenmeyer flasks on an orbital shaker and were inoculated with 5% (v/v) from a well-grown preculture. Growth of cells was measured photometrically in a Klett-Summerson photometer (Manostat) using filter no. 54 (520-580 nm). Samples were withdrawn depending on the growth phase from each culture in the exponential (Figure 1a: 385-440 Klett units (KU), Figure 1b: 300-400 KU), the early stationary and the stationary growth phase, and were quantified for their polyester contents by gas chromatography analysis. After samples had been withdrawn in the early stationary phase, NH_4_Cl was added to the cultures to a final concentration of 0.05% (w/v) to induce PHB degradation. All samples of the stationary growth phase were withdrawn 6 h after induction with ammonium chloride.

**Table 2:** Bacterial strains and mutants used in this study

Strain	Description	Reference or source
*Ralstonia eutropha*		
H16	Wild type	DSM 428
HF39	Sm^r ^strain of the wild type H16	Srivastava et al. 1982
PHB^-^4	PHB-negative mutant of the wild type H16	DSM 541
Δ*ptsM*	*ptsM *precise deletion gene replacement mutant of strain H16	Kaddor and Steinbüchel 2011
Δ*ptsH*	*ptsH *precise deletion gene replacement mutant of strain H16	Kaddor and Steinbüchel 2011
Δ*ptsI*	*ptsI *precise deletion gene replacement mutant of strain H16	Kaddor and Steinbüchel 2011
Δ*ptsHI*	*ptsHI *precise deletion gene replacement mutant of strain H16	Kaddor and Steinbüchel 2011
Δ*ptsMHI*	*ptsMHI *precise deletion gene replacement mutant of strain H16	Kaddor and Steinbüchel 2011
ΔH16_A2203	H16_A2203 precise deletion gene replacement mutant of strain H16	Kaddor and Steinbüchel 2011
Δ*ptsH *ΔH16_A2203	*ptsH*, H16_A2203 precise deletion gene replacement mutant of strain H16	Kaddor and Steinbüchel 2011
Δ*frcACB*	*frcACB *precise deletion gene replacement mutant of strain H16	Kaddor and Steinbüchel 2011
Δ*ptsMHI *Δ*frcACB*	*ptsMHI, frcACB *precise deletion gene replacement mutant of strain H16	Kaddor and Steinbüchel 2011
Δ*nagFEC*	*nagFEC *precise deletion gene replacement mutant of strain H16	Kaddor and Steinbüchel 2011
Δ*ptsMHI *Δ*nagFEC*	*ptsMHI*, *nagFEC *precise deletion gene replacement mutant of strain H16	Kaddor and Steinbüchel 2011
ΔH16_A0384	H16_A0384 precise deletion gene replacement mutant of strain H16	Kaddor and Steinbüchel 2011
Δ*ptsMHI *ΔH16_A0384	*ptsMHI*, H16_A0384 precise deletion gene replacement mutant of strain H16	Kaddor and Steinbüchel 2011
*ptsI*::Tn*5*::*mob*	strain HF39 with Tn*5*-inertion in *ptsI*, Sm^r ^Km^r^	Pries et al. 1991; Schubert et al. 1988
*ptsMH*::Tn*5*::*mob*	strain HF39 with Tn*5*-insertion in *ptsM-ptsH*, Sm^r ^Km^r^	Kaddor and Steinbüchel 2011

### PHB analysis

Lyophilized cell material of *R. eutropha *(5-10 mg) was subjected to methanolysis in presence of 85% (v/v) methanol and 15% (v/v) sulfuric acid for 3 h at 100°C. The resulting methyl esters of 3-hydroxybutyrate were characterized by gas chromatography as described previously (Brandl et al. 1988; Timm and Steinbüchel 1990) by using an Agilent 6850 GC (Agilent Technologies, Waldbronn, Germany) equipped with a BP21 capillary column (50 m by 0.22 mm; film thickness, 250 nm; SGE, Darmstadt, Germany) and a flame ionization detector (Agilent Technologies).

## Results

### Growth behavior of mutants in liquid media containing glycerol

As the most obvious result of these experiments two groups of mutants with different growth and accumulation behavior were revealed. Figure 1 summarizes the results of the cultivation experiments in MSM containing glycerol.

The first group A (Figure 1a) is represented by the wild type H16 whose increase of optical density ceased after 150 h of cultivation and exhibited a maximum optical density of 720 KU (350 h). Mutants H16 ΔH16_A2203, H16 ΔH16_A0384, H16 Δ*ptsMHI *ΔH16_A0384, H16 Δ*frcACB *and H16 Δ*nagFEC *belonging to group A behaved similarily like the wild type.

The second group B, Figure 1b represented by the PHB-negative mutant PHB^-^4 exhibited growth curves with the lowest optical density when the cells entered the early stationary growth phase after 100 h of cultivation comprising only about 50% of the optical density of the wild type strain H16 (about 380 KU). The apparent slowest growth of strain PHB^-^4 is due to its inability to accumulate PHB in form of intracellular granules; the latter contribute to the optical density of a culture. After addition of NH_4_Cl to the PHB^-^4 culture and to the other strains belonging to this group, the optical densities of these cultures increased, in case of PHB^-^4 up to 550 KU and in case of H16 Δ*ptsM*, a mutant lacking the fructose-specific EIIA^Man ^component, up to 660 KU. Group B is related to the PHB^-^4 characteristics and comprises mutants H16 Δ*ptsM*, H16 Δ*ptsH*, H16 Δ*ptsI*, H16 Δ*ptsMHI*, H16 Δ*ptsH *ΔH16_A2203, H16 Δ*ptsMHI *Δ*frcACB*, H16 Δ*ptsMHI *Δ*nagFEC*, as well as the two transposon-induced mutants HF39 *ptsMH*::Tn*5*::*mob *and HF39 *ptsI*::Tn*5*::*mob*.

### PHB accumulation of mutants utilizing glycerol as a sole carbon source

**Figure 2:**
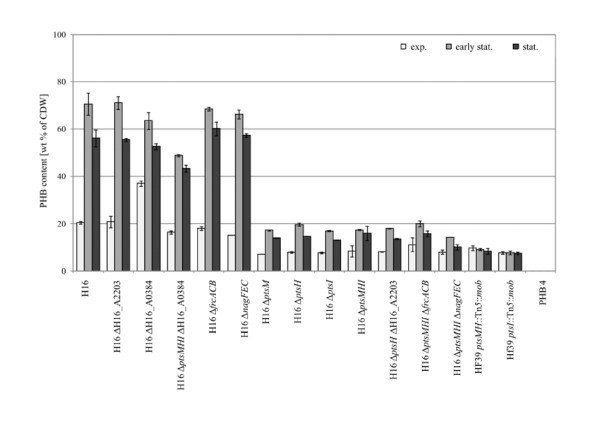
**PHB accumulation by *R. eutropha *H16 and various mutants**. Samples were withdrawn in the exponential (light gray bars), early stationary (gray bars) and stationary (dark gray bars) growth phases of cultivation and were analyzed by gas chromatography. Data are mean values of two independent experiments ± standard deviations.

The intracellular accumulation of PHB found in mutants of *R. eutropha *grown in MSM with glycerol as a sole carbon source is shown in Figure 2. The left side of the figure shows the results obtained with mutant strains belonging to group A of Figure 1, whereas the right side comprises the results obtained with mutant strains belonging to group B of Figure 1.

In comparison to accumulation experiments made in MSM plus sodium gluconate or fructose (Kaddor and Steinbüchel 2011), the capability of some mutants to accumulate PHB was reduced up to 24% (w/w) of cell dry matter when cells were cultivated in MSM containing glycerol. This may be due to the limited number of available carbon and to the competition of PHB biosynthesis and gluconeogenesis for C_3_-intermediates required for product formation and growth (Bormann and Roth 1999). As expected, strain H16 synthesized large amounts of PHB (70.7%, w/w) in the early stationary growth phase (95 h), whereas strain PHB^-^4 did not accumulate any detectable polyester at all. In contrast, Chee et al. (2010) obtained only about 33% (w/w) PHB in the cells during cultivation of the wild type H16 in modified MSM with glycerol as a sole carbon source for 72 h.

Mutant strains belonging to group A stored PHB in the range of 49-71.3% (w/w) at the maximum, whereas in strains belonging to group B PHB contents of 20.1% (w/w) were not exceeded. In this group, the lowest PHB contents were obtained for the Tn*5*-induced mutant HF39 *ptsI*::Tn*5*::*mob *which seemed not to enhance PHB biosynthesis in the early stationary phase (7.8%, w/w). Moreover, the strain did not degrade any PHB after induction with ammonium chloride although the optical density increased after this time (Figure 1b). Mutant HF39 *ptsMH*::Tn*5*::*mob *behaved similarly to this mutant which implies that the inserted Tn*5 *affected synthesis as well as mobilization of PHB. This observation was not made when sodium gluconate, fructose or *N*-acetylglucosamine were used as carbon source (Kaddor and Steinbüchel 2011).

## Discussion

When comparing group A with group B, it is noticeable that the deletion of *ptsM*, *ptsH*, or *ptsI *exerted a significant change of the PHB synthesis phenotype. Besides the Tn*5*-induced mutants, the remaining mutants of group B harbored in addition the deletion of either *ptsM*, *ptsH*, *ptsI *or all three genes. Another mutant, H16 Δ*ptsHI *behaved similar like mutant H16 Δ*ptsMHI *(data not shown). The impact of the putative *ptsMHI *operon was observed during growth in presence of both, the PTS carbohydrate *N*-acetylglucosamine and the non-PTS carbon sources sodium gluconate, fructose, and glycerol. Particularly, during growth on glycerol in comparison to growth on the previously analyzed carbon sources (Kaddor and Steinbüchel 2011), mutants defective in the putative *ptsMHI *operon accumulated less PHB than the wild type. Pries et al. (1991) made similar observations with Tn*5*-induced *ptsH*/*ptsI *mutants exhibiting a PHB-leaky phenotype with a lower PHB content of the cells when grown on gluconate. However, a faster mobilization of PHB after exhausting the extracellular carbon source, as it occurred in presence of gluconate, was not noticed when cultivated in media containing glycerol. Despite the still unknown functions of *ptsH *and *ptsI*, an exclusively regulatory role in PHB and carbon metabolism was already proposed (Pries et al. 1991; Kaddor and Steinbüchel 2011).

Additionally, our study gives evidence for the involvement of *ptsM *(fructose-specific EIIA^Man^) in this regulatory mechanism, indicating a functional *ptsMHI *operon which is supported by the corresponding gene organization. It has already been proven that PtsM is not involved in fructose uptake and transport (Kaddor and Steinbüchel 2011), and therefore, the relation to EIIA^Man ^remained undetermined. Mutant H16 ΔH16_A0384 lacking the nitrogen regulatory EIIA^Ntr ^component did not show PHB overproduction in MSM plus glycerol (63.6%, w/w, PHB) as it was observed during growth in MSM plus gluconate (87.6%, w/w, PHB). As it is obvious from the quadruple mutant H16 Δ*ptsMHI *ΔH16_A0384 (49%, w/w, PHB), the high decrease of PHB production in the triple mutant H16 Δ*ptsMHI *(17.5%, w/w, PHB) seemed to be compensated by the additional deletion of H16_A0384 that has also been observed during cultivation experiments with the non-PTS sugars sodium gluconate or fructose as carbon source (Kaddor and Steinbüchel 2011). In disruption mutants of *Azotobacter vinelandii *UW136, RN7 (*ptsN*::Km^r ^*ptsP*::Tc^r^) and RN8 (*ptsN*::Km^r ^*ptsO*::Sp^r^), the negative effect of the single *ptsP *or *ptsO *mutation on PHB accumulation was suppressed in the double mutants as well (Noguez et al. 2008). The same result was obtained for a *Pseudomonas putida *MAD2 double mutant (*ptsN*::*xylE ptsO*::Km^r^) (Velázquez et al. 2007). Unlike the mutation of ΔH16_A0384, the deletion of H16_A2203 (HPr-related phosphocarrier protein), *frcACB *(fructose-specific ABC-type transporter) or *nagFEC *(PTS^Nag^) could not enhance the growth and limited PHB accumulation of the derived multiple mutants H16 Δ*ptsH *ΔH16_A2203, H16 Δ*ptsMHI *Δ*frcACB *and H16 Δ*ptsMHI *Δ*nagFEC*.

The limited carbohydrate utilization range of *R. eutropha *coupled with the high costs of these carbon sources in biopolymer production restricts its application in biotechnological processes. Renewable substitutes for the so far used expensive substrates must be investigated to lower the commercial PHA production costs (e.g. of the thermoplastic Biopol) to make them competitive with the petrochemical-based plastic manufacture. Based on the experimental results, it appears that polymer accumulation in strain H16 is reduced to a minor extend when cells were grown on glycerol (70.7%, w/w, PHB after 260 h of cultivation) in comparison to accumulation experiments made on sodium gluconate or fructose (up to 78%, w/w, PHB after 28 h of cultivation, Kaddor and Steinbüchel 2011). We demonstrated that strain H16 has the potential to utilize glycerol for indeed suboptimal growth but with an unrestricted capability of valuable PHB production. Although glycerol transport and utilization is independent of the PEP-PTS in strain H16, deletion of the PTS homologous genes affected anyhow carbon and PHB metabolism in this strain indicating a complex regulatory function of the PTS. However, the slow growth of the wild type on this cheap and abundant sugar substitute prevents it currently from its use in industrial large-scale productions. Heterotrophic growth on glycerol is related to carbon and energy limiting conditions. Besides oxidative stress proteins and hydrogenases, enzymes of gluconeogenesis and the CBB pathway as well as PhaA and PhaB belong to the most abundant proteins of glycerol-grown cells (Friedrich et al. 1981; Schwartz et al. 2009).

This study focussed on the involvement of PTS homologous proteins on the utilization of glycerol with respect to polymer biosynthesis in *R. eutropha *H16. Four PTS homologous proteins (PtsM, PtsH, PtsI, PtsN) showed a significant influence during glycerol utilization on both, cell growth and PHB accumulation. Deletion of the fructose-specific transport proteins resulted in no significant difference to the wild type concerning growth and storage behavior. Due to the occurrence of PEP-PTS homologous proteins and the absence of a PTS-mediated carbohydrate uptake in this strain except for the PTS^Nag^, further investigations are required to unravel their functions in this PHB producing strain. Besides the generation of deletion mutants and their phenotypical characterization, intensive studies on the putative operon *ptsMHI *are now necessary to characterize the respective genes in more detail and to resolve their roles in the metabolism of *R. eutropha*. Certainly, this study provides a further degree of regulation between the general PTS proteins and both, PHB and carbon metabolism in *R. eutropha *H16.

## Competing interests

The authors declare that they have no competing interests.
